# Transcriptional responses of liver and spleen in *Lota lota* to polyriboinosinic polyribocytidylic acid

**DOI:** 10.3389/fimmu.2023.1272393

**Published:** 2023-10-13

**Authors:** Fangrui Lou, Yuan Zhang, Anle Xu, Tianxiang Gao

**Affiliations:** ^1^ School of Ocean, Yantai University, Yantai, Shandong, China; ^2^ CAS Key Laboratory of Tropical Marine Bio-resources and Ecology, South China Sea Institute of Oceanology Chinese Academy of Sciences, Guangzhou, China; ^3^ Fishery College, Zhejiang Ocean University, Zhoushan, Zhejiang, China

**Keywords:** *Lota lota*, liver, spleen, transcriptome, poly (I:C), viral response mechanism

## Abstract

**Introduction:**

The cultured *Lota lota* can meet the market demand in the context of the decline of wild resources, but the disease in the high-density culture process also deserves attention. Therefore, understanding the immune regulation mechanisms of *L. lota* will be the basis for obtaining high benefits in artificial culture.

**Methods:**

To explore the viral response mechanism of *L. lota*, RNA-seq was applied to identify the transcriptomic changes of the liver and spleen in *L. lota* by poly (I:C) stress.

**Results:**

The DEGs (liver: 2186 to 3123; spleen 1542 to 2622) and up-regulated genes (liver: 1231 to 1776; spleen 769 to 1502) in the liver and spleen increased with the prolongation (12h to 48h) of poly (I:C)-stimulation time. This means *L. lota* needs to mobilize more functional genes in response to longer periods of poly (I:C)-stimulation. Despite the responses of *L. lota* to poly (I:C) showed tissue-specificity, we hypothesized that both liver and spleen of *L. lota* can respond to poly (I:C) challenge may be through promoting apoptosis of DNA-damaged cells, increasing the activity of immune-enhancing enzymes, and increasing energy supply based on DEGs annotation information.

**Conclusions:**

Our results demonstrate the transcriptional responses of *L. lota* to poly (I:C)-stimulation, and these data provide the first resource on the genetic regulation mechanisms of *L. lota* against viruses. Furthermore, the present study can provide basic information for the prevention of viral diseases in *L. lota* artificial culture process.

## 1 Introduction

Viruses are transmitted horizontally into aquatic fish mainly through the gills and intestine, causing oxidative damage, dysfunction, inflammation, and even death (1, 2). Because aquatic fish do not have perfect antiviral capacity (3), the outbreak of the virus will lead to loss of control of aquatic fish management (4).

The immune organs of aquatic fish are mainly composed of head-kidney, liver, spleen, and so on (5, 6). Among them, the head-kidney can produce red blood cells and B lymphocyte without relying on antigen stimulation, and thus has the dual function of central and peripheral immune organ. Meanwhile, the head-kidney can not only store, destroy, and detoxify various foreign matter, but also participate in the inflammatory response and humoral immune process as the primary center of memory cells (5, 7). The liver has been shown to contain a variety of natural immune cells that can induce immune tolerance or inflammatory responses and produce various cytokines and chemokines (8, 9). Previous studies have suggested that the liver can also synthesize complement (10, 11). Tafalla et al. (12) revealed that the spleen of aquatic fish also had immune function, and its role in non-specific and specific immune systems was only weaker than that of the head-kidney. Although the functions of melano-macrophages in the spleen have not been determined, the virus-clearing function of melano-macrophages is well established and they may be the lymphocyte germinal center (13). Consequently, investigating the immune responses of the immune organs to invading viruses can demonstrate how the fish cope with viral infection.

*Lota lota* is the only freshwater Gadiformes and is widely distributed in inland lakes and bays of Europe, Asia, and North America north of latitude 45°N (14). Climate warming caused by human activities and other factors is assumed to have resulted in a rapid decrease of *L. lota* resources, which are ecologically dependent on low temperature (15, 16), and *L. lota* has been listed in the Rare Aquatic Wildlife of China. The gradual development of artificial culture can meet the market demand for aquatic food under the background of the decline of *L. lota* resources (17). Parasitic and viral infections have seriously limited the health and sustainable development of *L. lota* aquaculture (18), but there is little research on how to deal with these problems.

Transcriptional processes usually represent the complex dynamics of biological internal regulatory mechanisms (16), which can be used to reveal the response mechanisms of *L. lota* to invading viruses. High-throughput sequencing technologies such as RNA sequencing (RNA-seq) have advantages in obtaining complete transcripts of organisms (19), and thus have been successfully applied to analyze the transcriptional responses of a variety of aquatic fishes to viral invasion (20–22). Therefore, RNA-seq can also serve as a useful technique for identifying the transcriptional divergences of *L. lota* associated with viral invasion. Unfortunately, transcriptome studies of *L. lota* have only focused on the response mechanisms of high temperature (16, 23), and the transcriptional regulation mechanisms of *L. lota* in response to viral invasion have not been reported.

Polyriboinosinic polyribocytidylic acid [poly (I:C)] is the viral double-stranded RNA analogue that as an immune adjuvant has been shown to induce viral immune responses in fishes or shellfishes (22, 24–26). In the present study, the liver and spleen of *L. lota* injected with poly (I:C) were obtained and RNA-seq was carried out to explore the genetic regulation mechanisms of *L. lota* on poly (I:C). Furthermore, our results can provide new insights into the immune regulation of *L. lota* against viruses, and provide basic data for viral disease prevention in the *L. lota* intensive culture processes.

## 2 Materials and methods

### 2.1 Ethics approval and participation consent

We promise that the present research complies with the applicable international and institutional policies relating to animal experiments. Meanwhile, all experimental methods involved in this study were approved by the Institutional Animal Care and Use Committee of Yantai University and performed in accordance with relevant guidelines and regulations. Additionally, all *L. lota* used in the present study were quickly transported to the laboratory and then were anesthetized with tricaine methanesulfonate (100 mg/L) before dissection.

### 2.2 *L. lota* collection, maintenance, and poly (I:C) stimulation

*L. lota* (28.2 ± 1.03 g, 15.6 ± 0.90 cm) were collected from the aquafarm of the Irtysh River, Burqin, Xijiang Province (China) on 29 October 2019. Before poly (I:C) stimulation, all *L. lota* were placed temporarily in the same polyethylene aquariums (volume: 40 L) for a week to acclimatize, and the temperature, pH, ammonia nitrogen, nitrite, nitrate, and dissolved oxygen of water were maintained at 17°C, 7.9, 0.27 mg/L, 0.001 mg/L, 0.05 mg/L, and 5.0 mg/L, respectively. Normal feeding (3% of body weight; at 08:00 and 14:00) was performed throughout the experimental process. Meanwhile, we change the water every morning to ensure the stability of all water factors. After acclimation, all *L. lota* were randomly assigned to two separate aquariums (length × width × height: 72 cm × 53 cm × 44 cm), each of which was assigned 10 individuals (6 were used for subsequent experiments and the remaining 4 were used as backup), and the breeding density of *L. lota* was approximately 1,679.57 g/m^3^. We intraperitoneally injected 200 μL of PBS reagent (Beyotime Biotechnology, Shanghai, China) into individual *L. lota* in an aquarium as the control group, and injected 200 μL of poly (I:C) (Beyotime Biotechnology, Shanghai, China) at a concentration of 0.5 mg/mL (dissolved using PBS reagent) into individual *L. lota* in another aquarium as the treatment group. At 12 h and 48 h after injection, *L. lota* in the two groups were sacrificed and dissected to obtain the liver and spleen, and three individuals were randomly collected as biological replicates at each time point in each group. The obtained livers and spleens were immediately frozen with liquid nitrogen and then stored at −80°C. Finally, 24 tissue samples (2 tissues × 2 groups × 2 time nodes × 3 biological replicates) were obtained for subsequent RNA extraction. All experimental groups were named L_12PBS_, S_12PBS_, L_12po_, S_12po_, L_48PBS_, S_48PBS_, L48_po_, and S_48po_, respectively.

### 2.3 RNA extraction and detection, library construction, and Illumina sequencing

We used the Trizol Reagent Kit (Vazyme, Nanjing, China) to extract the total RNA of each tissue sample following the manufacturer’s protocol. The degradation or contamination degree, purity (OD260/280), concentration, and integrity of RNAs was detected using the agarose gel electrophoresis, Nanodrop (Implen, MUC, Germany), Qubit 2.0 (Thermo Fisher Scientific, MA, USA), and Agilent 2100 (Agilent Technologies, CA, USA), respectively.

The qualified total RNAs were used for library construction. We removed rRNA from the 1 μg of total RNA per sample and purified the remaining mRNA using RNA Purification Beads (Illumina, San Diego, CA, USA), and then cleaned all mRNAs three times using the Beads Binding Buffer (Illumina, San Diego, CA, USA). We used the NEBNext® UltraTM RNA Library Prep Kit for Illumina ® (NEB, USA) to construct 24 sequencing libraries after mRNA fragmentation. Specifically, we first reversely transcribed the mRNA into first-strand cDNA using random hexamers, and then added buffer, dNTPs, and DNA polymerase I to synthesize the second-strand cDNA. The synthesized double-stranded cDNAs were purified using AMPure XP beads (Illumina, San Diego, CA, USA), and terminal repair, A-tail addition, and sequencing connector were then performed. Subsequently, we added A-tails and adapters to the double-stranded cDNAs, respectively. Then, AMPure XP Beads were applied to select the fragment size, and these fragments with appropriate size were amplified by PCR to obtain the cDNA libraries. The constructed cDNA libraries were quantified using Qubit 2.0 (Thermo Fisher Scientific, MA, USA) and then diluted to 1 ng/µL. Finally, the cDNA libraries qualified by the Agilent 2100 (Agilent Technologies, CA, USA) were sequenced on an Illumina HiSeq 2000 platform across one lane with 150 bp paired-end.

### 2.4 RNA-seq data processing

Trimmomatic software (version 0.36, 27) was used to eliminate low-quality raw RNA-seq reads, mainly referring to these reads that contained sequencing adapters, unknown nucleotides ratio > 10%, and quality scores < 20. Hisat software (version 2.0.4; 28) was used to compare the high-quality clean RNA-seq reads to the published *L. lota* whole genome sequences (29). The regional distribution and density distribution of clean RNA-seq reads on the genome sequences were then analyzed. Meanwhile, we use rMATS (version 3.2.5; 30) software to predict the classification, number, and structure of alternative splicing (AS) events.

### 2.5 Transcriptional responses of liver and spleen in *L. lota* to the poly (I:C) stimulation

To identify the transcriptional responses of liver and spleen in *L. lota* to the poly (I:C) stimulation, we first quantified the gene expression levels of each sample using the HTSeq (version 2.0; 31) software and described them using FPKM (expected number of Fragments Per Kilobase of transcript sequence per Millions base pairs sequenced). Then, we identified the differentially expressed genes (DEGs) among different experimental groups using DESeq software (version 2.0, 32), and false discovery rate (FDR) adjusted *p*-value < 0.005 and fold change (FC) ≥ 2 (which corresponds to |log_2_FC| ≥ 1) were used as the filtering thresholds. A Venn diagram was applied to visualize the number of DEGs. Furthermore, we determined the clustering relationship of DEG expression level in four experimental groups according to the FPKMs. Finally, we attempted to analyze the biological functions of these DEGs and their products based on the Gene Ontology (GO) and Kyoto Encyclopedia of Genes and Genomes (KEGG) annotation. The hyper-geometric distribution corrected *p*-value < 0.05 was taken as the criterion of significant enrichment of the GO term and KEGG pathway.

### 2.6 Quantitative reverse transcription PCR validation

In the present study, quantitative reverse transcription PCR (qRT-PCR) was applied to verify the reliability of transcriptome data. According to the functional annotation information, a total of eight immune-related DEGs (four in the liver and four in the spleen) were randomly selected for qRT-PCR experiments. Meanwhile, Ribosomal protein S29 (*rps29*) and Ribosomal protein L26 (*rpl26*) were used as reference genes for standardization (22). Primer Premier 6.0 was applied to design the reference gene-specific and DEG-specific primers (**Table 1**). Twenty-four cDNA samples from 12 experimental individuals were diluted 25 times using nuclease-free water according to standard curves and then used as templates for qRT-PCR. The qRT-PCR was conducted on the StepOne Plus Real-Time PCR system (ABI, USA) according to the manufacturer’s instructions of the TaKaRa TB Green Premix Ex Taq (Til RAaseH Plus, RR420A). A 20-μL reaction system was formulated, including 2 μL of diluted cDNA template, 0.4 μL of forward primer (10 μM), 0.4 μL of reverse primer (10 μM), 0.4 μL of ROX Reference Dye (50×), and 6.8 μL of RNase-free water. The qRT-PCR cycling conditions were as follows: one cycle of 95 °C for 30 s, followed by 40 cycles of 5 s at 95°C, 30 s at 60°C, and then enter the dissociation stage. Three experimental triplicates were performed for each qRT-PCR cycling condition to ensure the accuracy of qRT-PCR results. The relative expression levels of eight DEGs were calculated by the 2^−ΔΔCT^ method (ΔCT = CT_DEG_ − CT_internal gene_, ΔΔCT = ΔCT_treatment group_ – ΔCT_control group_).

**Table 1:** Primer sequences of 2 reference genes and 12 DEGs.

	Gene name	Full gene name	Primer (5′ to 3′)	Product length
Reference genes	*rps29*	Ribosomal protein S29	For_ ACAGCTCTACTGGAGTCAT	146 bp
			Rev_ CGAAGCCGATGTCCTTAG	
	*rpl26*	Ribosomal protein L26	For_ GCAAGAGGCACTTCAATG	127 bp
			Rev_ ACCTGGACTTCGTCATCT	
DEGs in the liver	*met*	Hepatocyte growth factor	For_GCCACATACAGGTTCTCTT	210 bp
			Rev_ACGACAGCAGACAGGAA	
	*casp3*	Caspase 3	For_CTGTGCGAGATGCTGAC	143 bp
			Rev_GTGGTGATGGCTGGAATC	
	*hsp90a1*	Heat shock protein 90 class A member 1	For_CGAGAAGAAGAAGCAGGAT	104 bp
			Rev_GGTTGGAGACGGAGACT	
	*lgp2*	Laboratory of genetics and physiology 2	For_GGTGGTGGTCCTGGTTA	217 bp
			Rev_AGCGTGTCTGTCCTCAT	
DEGs in the spleen	*pmp22*	Peripheral myelin protein 22	For_TTCTTCTTGGAGTGCTTGT	159 bp
			Rev_CATTGCTGGCTGGTAGG	
	*csf1r*	Colony stimulating factor 1 receptor	For_CGTGGTGGATGCTAACTT	177 bp
			Rev_TCATTGGTGGAGAGGAGAT	
	*ccnd2*	Cyclin-D2	For_CTACACAGACAACTCCATCA	128 bp
			Rev_GCAGCCTCCTCACAATG	
	*flt3*	FMS-like tyrosine kinase 3	For_ACAACGACTCCAACTACG	271 bp
			Rev_CTCCAGCACTTACACATCA	

## 3 Results

### 3.1 RNA-seq data of 24 samples

**Figure 1:**
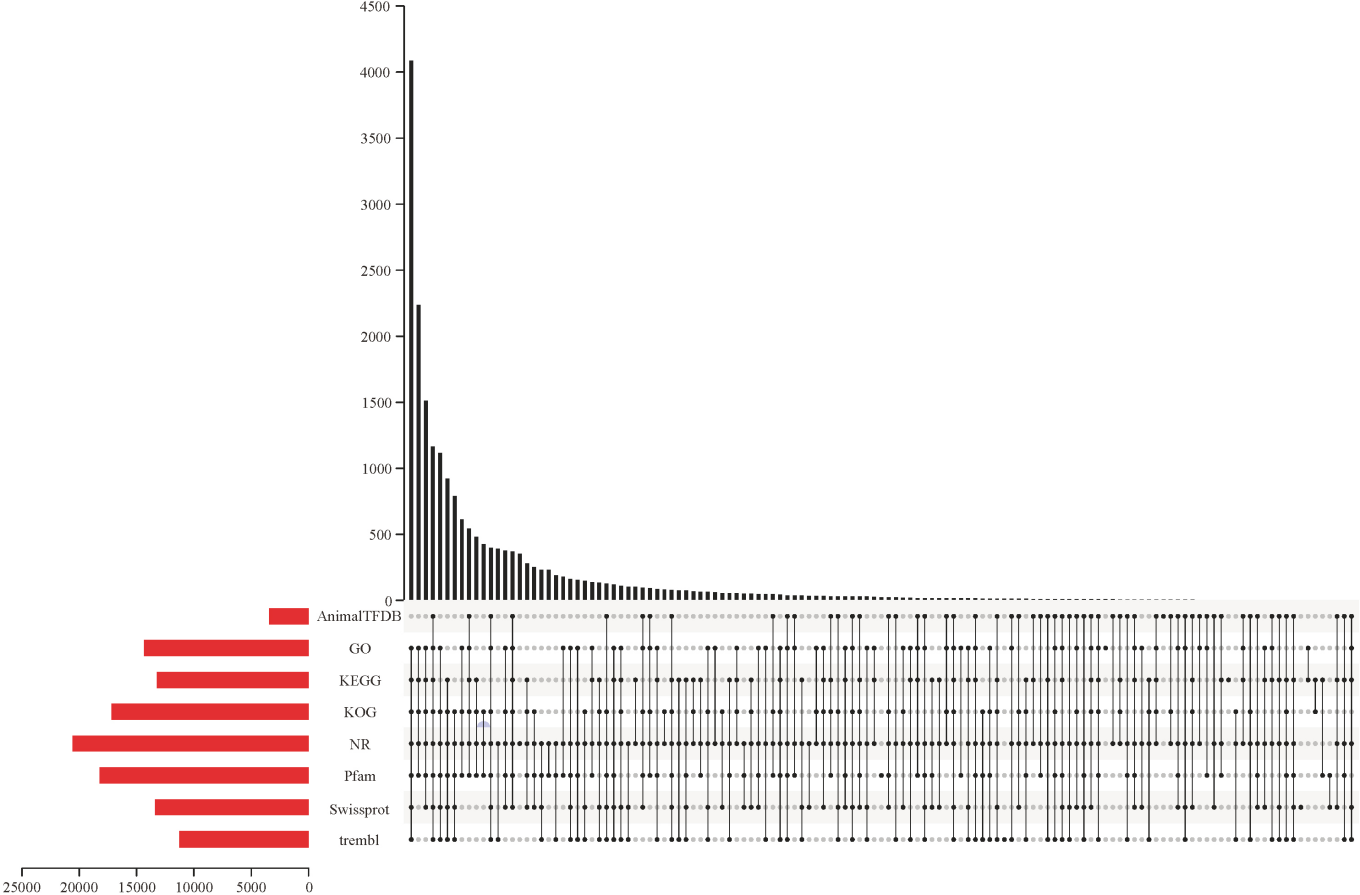
The number of genes successfully matched to sequences in the AnimalTFDB, GO, KEGG, KOG, NR, Pfam, Swiss-Prot, and TrEMBL databases, respectively.

All raw RNA-seq reads have been submitted to the NCBI sequence read archive under the BioProject PRJNA874751. A total of 228.08 Gb of clean RNA-seq reads were captured after removing the low-quality raw RNA-seq reads, and the summary of RNA-seq reads is shown in **Supplementary File 1**. All clean RNA-seq reads from 24 samples were compared to the reference genome (**Supplementary File 2**) and results showed that 90.97% of the clean RNA-seq reads can be located on the reference genome. Meanwhile, the percentages of clean RNA-seq reads with multiple mapped locations and uniquely mapped locations on the reference genome are 7.14% and 83.83%, respectively. Furthermore, although many clean RNA-seq reads are mapped to the exon region of reference genome, a small number of reads are mapped to the intergenic region, and a minimal proportion of clean RNA-seq reads were compared to intron regions (**Supplementary File 3**). Meanwhile, there was a positive correlation between the distribution density of clean RNA-seq reads and chromosome length (**Supplementary File 4**). Additionally, a total of 20,646 predicted genes were successfully annotated on at least one protein database. Of all annotated predicted genes, 3,432, 14,369, 13,246, 17,187, 20,576, 18,218, 13,388, and 11,282 predicted genes had significantly matched with the sequences in the AnimalTFDB, GO, KEGG, KOG, NR, Pfam, Swiss-Prot, and TrEMBL databases, respectively (**Figure 1**).

### 3.2 AS events

A total of 3,347 and 3,361 AS events were predicted based on “junction count only-” and “reads on target and junction counts-” quantitative methods, respectively. Meanwhile, a total of 198 and 202 different AS events were predicted based on “junction count only-” and “reads on target and junction counts-” quantitative methods, respectively. Among the five common AS events (namely, skipped exon [SE], alternative 5′ splice site [A5SS], alternative 3′ splice site [A3SS], mutually exclusive exons [MXE], and retained intron [RI]), SE and MXE were found to be the most abundant AS events (**Table 2**).

**Table 2:** AS classification and quantitative statistics.

	SE	MXE	A5SS	A3SS	RI
Number of ASs based on junction count only	3,161	186	0	0	0
Number of different ASs based on junction count only	178 (81:97)	20 (4:16)	0	0	0
Number of ASs based on reads on target and junction counts	3,175	186	0	0	0
Number of different ASs based on reads on target and junction counts	181 (82:99)	21 (4:17)	0	0	0

### 3.3 The number of DEGs of *L. lota* stimulated by poly (I:C)

**Figure 2:**
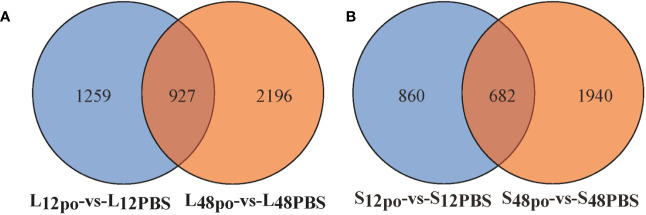
The poly (I:C)-stimulated DEGs in liver **(A)** and spleen **(B)** of *L. lota*.

**Figure 3:**
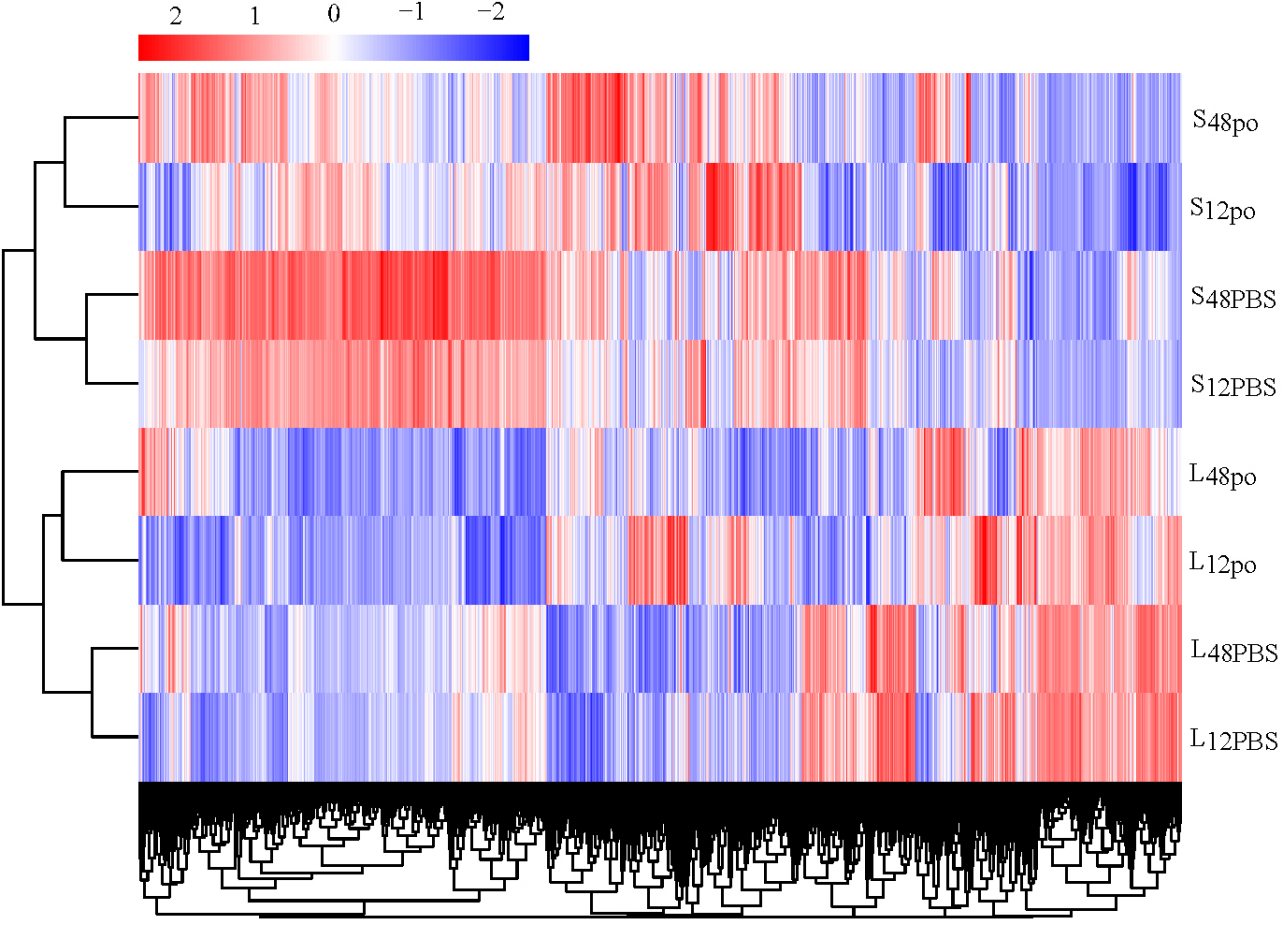
The clustering relationship of DEG expression levels of four experimental groups according to the FPKM.

In the present study, we have identified the poly (I:C)-stimulated DEGs in liver (L_12po_-vs-L_12PBS_, L_48po_-vs-L_48PBS_) and spleen (S_12po_-vs-S_12PBS_, and S_48po_-vs-S_48PBS_) of *L. lota* (**Supplementary File 5**). Results showed that the number of DEGs increased with the increase in infiltration time of liver (2,186 at 12 h and 3,123 at 48 h; **Figure 2A**) and spleen (1,542 at 12 h and 2,622 at 48 h; **Figure 2B**) in poly (I:C). Compared with the PBS treatments, a total of 1,231 (56.31%) and 1,776 (56.87%) upregulated genes were identified from the liver exposed to poly (I:C) at 12 h and 48 h, respectively. Meanwhile, a total of 769 (49.87%) and 1,502 (57.28%) upregulated genes were identified from the spleen exposed to poly (I:C) at 12 h and 48 h, respectively. Additionally, cluster analysis showed that the expression levels of DEGs from the same tissue and the same treatment were similar, and the similarity of DEG expression levels in the same tissue was higher than that between tissues (**Figure 3**).

**Figure 4:**
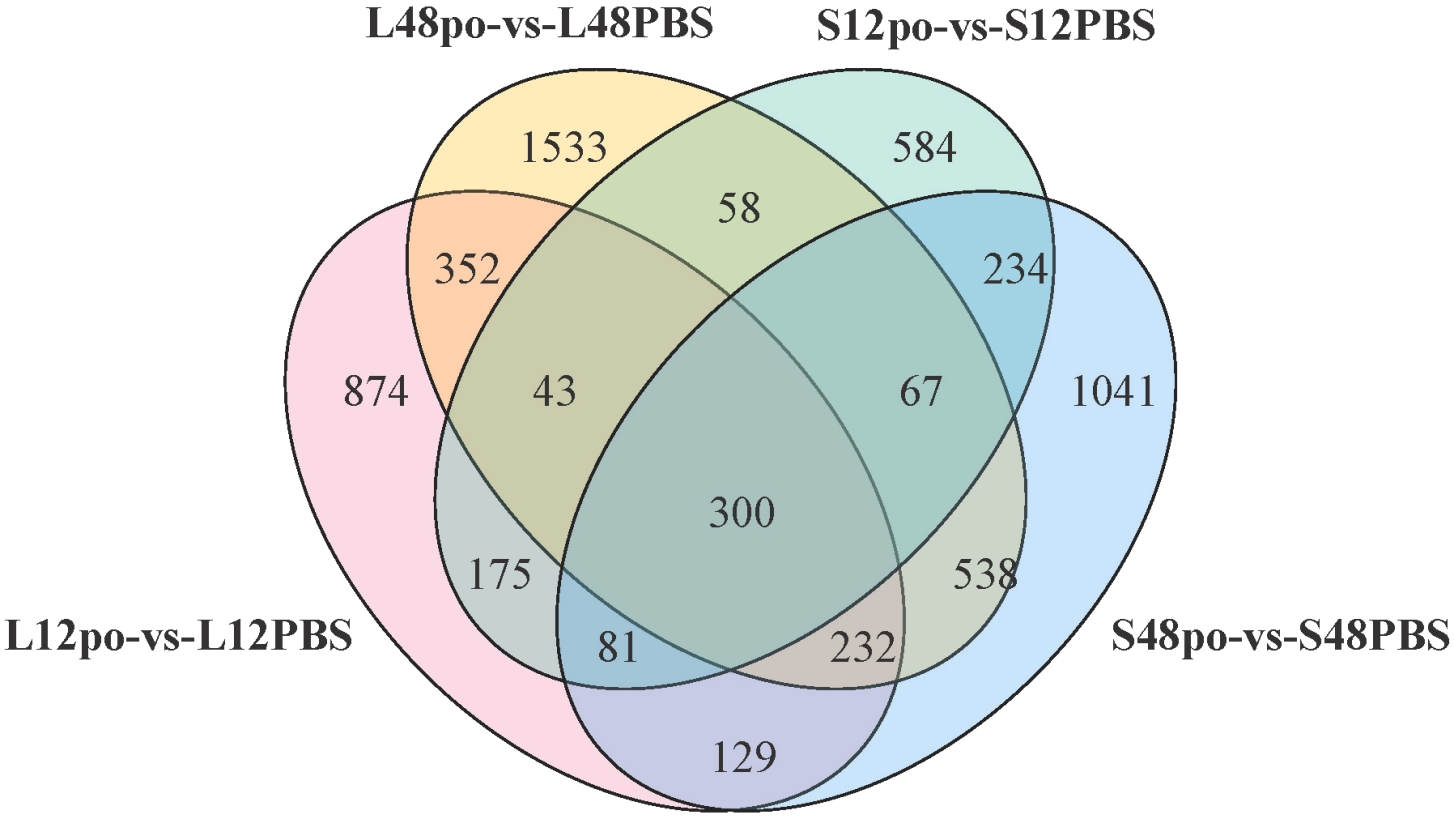
The common DEGs among four experimental pairs.

We found that a substantial overlap of 300 DEGs existed in four experimental pairs (L_12po_-vs-L_12PBS_, L_48po_-vs-L_48PBS_, S_12po_-vs-S_12PBS_, and S_48po_-vs-S_48PBS_) (**Figure 4**). Considering that these genes contributed more to the responses of *L. lota* to poly (I:C), we obtained annotation information of 300 DEGs (**Supplementary File 6**). Not unexpectedly, there are abundant immune-related genes (such as Nuclear valosin-containing protein [*NVLp*], Tumor necrosis factor [*TNF*], Zinc-binding protein [*ZBP*], Antigen peptide transporter (*APT*), Calcium/calmodulin-dependent protein kinase type 1G [*Camk1g*], and Interferon regulatory factor 4 [*IRF-4*]) in *L. lota* that were ultimately predicted (the yellow marked part of **Supplementary File 6**).

### 3.4 The biological functions of poly (I:C) response-associated genes in *L. lota*

**Figure 5:**
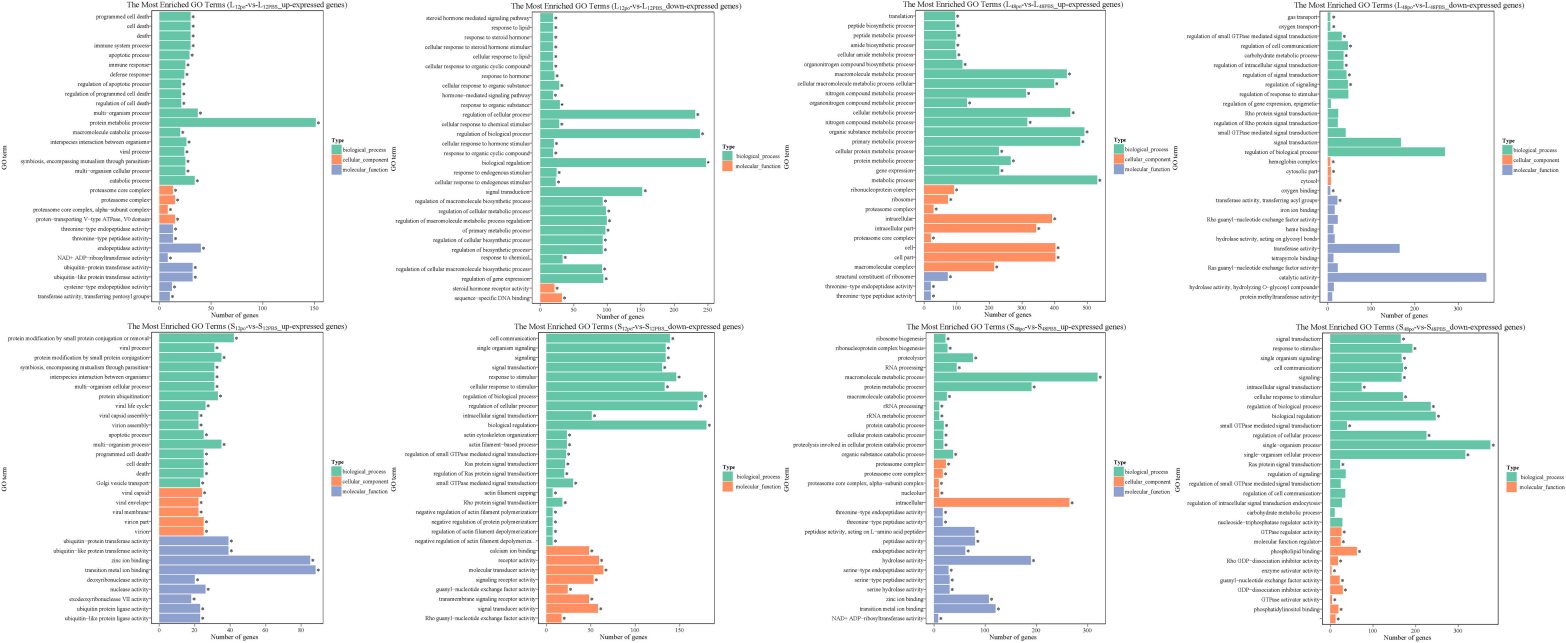
The top 30 significantly enriched GO terms of the up- and down regulated genes in the liver and spleen. The vertical coordinate represents the enriched GO terms, and the horizontal coordinate represents the number of DEGs in GO terms. Different colors are used to distinguish biological processes, cellular component, and molecular function. "*" represents the significance of GO terms.

Unsurprisingly, poly (I:C) injection significantly affected the biological processes of *L. lota*, and this phenomenon had time and tissue specificity. Firstly, we matched the up- and downreg ulated genes in the liver and spleen into the GO database and then obtained the GO annotation information (**Supplementary File 7**; **Figure 5**). At 12 h after poly (I:C) injection, upregulated genes in the liver and spleen were significantly enriched in 79 and 156 GO terms (corrected *p*-value < 0.05), respectively. Meanwhile, downregulated genes in the liver and spleen were significantly enriched in 78 and 74 GO terms (corrected *p*-value < 0.05), respectively. At 48 h after poly (I:C) injection, upregulated genes in the liver and spleen were significantly enriched in 162 and 73 GO terms (corrected *p*-value < 0.05), respectively. Meanwhile, downregulation in the liver and spleen were significantly enriched in 12 and 24 GO terms (corrected *p*-value < 0.05), respectively. The terms of up- or down regulated genes are related to protein regulation, amino acid regulation, energy regulation, cellular homeostasis, immune cell integrity and ion transport.

**Figure 6:**
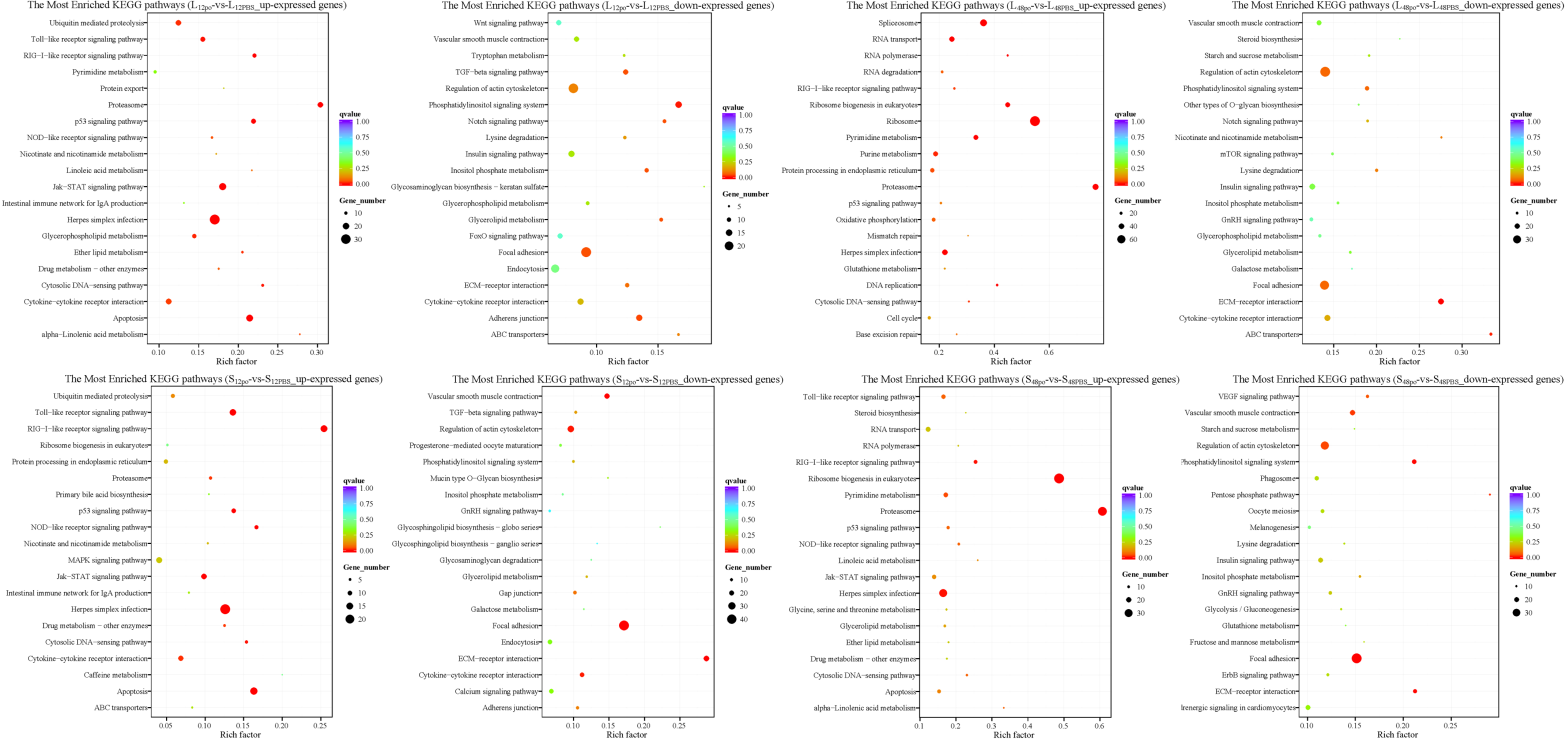
The top 20 significantly enriched KEGG pathways of the up- and down regulated genes in the liver and spleen. The vertical coordinate represents the enriched KEGG pathways, and the horizontal coordinate represents the Rich factor. The size of the dots represents the number of DEGs in KEGG pathways, and different colors of dots represent the different *q* values.3.5 Validation of immune-related gene expression levels.

We recorded the networks of molecular interactions in the cells and the variants specific to *L. lota* by mapping the up- and downreg ulated genes in the liver and spleen to the KEGG database, respectively (**Supplementary File 8**; **Figure 6**). At 12 h after poly (I:C) injection, upregulated genes in the liver and spleen were significantly enriched in the 14 (namely, Herpes simplex infection pathway, Proteasome pathway, Apoptosis pathway, Jak-STAT signaling pathway, p53 signaling pathway, RIG-I-like receptor signaling pathway, Toll-like receptor signaling pathway, Cytosolic DNA-sensing pathway, Glycerophospholipid metabolism, Ubiquitin-mediated proteolysis, Ether lipid metabolism pathway, Cytokine–cytokine receptor interaction pathway, alpha-Linolenic acid metabolism pathway, and NOD-like receptor signaling pathway) and 11 (namely, RIG-I-like receptor signaling pathway, Herpes simplex infection pathway, Apoptosis pathway, Toll-like receptor signaling pathway, p53 signaling pathway, NOD-like receptor signaling pathway, Jak-STAT signaling pathway, Cytosolic DNA-sensing pathway, Cytokine–cytokine receptor interaction pathway, Proteasome pathway, and Drug metabolism - other enzymes pathway) metabolic pathways, respectively (corrected *p*-value < 0.05). Meanwhile, downregulated genes in the liver and spleen were significantly enriched in the seven (namely, Phosphatidylinositol signaling system pathway, Adherens junction pathway, Focal adhesion pathway, Notch signaling pathway, Glycerolipid metabolism pathway, Inositol phosphate metabolism pathway, and TGF-beta signaling pathway) and five (namely, Focal adhesion pathway, ECM–receptor interaction pathway, Vascular smooth muscle contraction pathway, Regulation of actin cytoskeleton pathway, and Cytokine–cytokine receptor interaction pathway) metabolic pathways, respectively (corrected *p*-value < 0.05). However, the number and type of metabolic pathways significantly enriched at 48 h of poly (I:C) stimulation varied, which was present in both tissues. Results showed that upregulated genes in the liver and spleen were significantly enriched in the 13 (namely, Ribosome pathway, Proteasome pathway, Spliceosome pathway, Ribosome biogenesis in eukaryotes pathway, Pyrimidine metabolism pathway, RNA transport pathway, DNA replication pathway, Herpes simplex infection pathway, RNA polymerase pathway, Purine metabolism pathway, Cytosolic DNA-sensing pathway, RIG-I-like receptor signaling pathway, and Protein processing in endoplasmic reticulum pathway) and 5 (namely, Proteasome pathway, Ribosome biogenesis in eukaryotes pathway, Herpes simplex infection pathway, RIG-I-like receptor signaling pathway, and Pyrimidine metabolism pathway) metabolic pathways, respectively (corrected *p*-value < 0.05). Meanwhile, downregulated genes in the liver and spleen were significantly enriched in the two (namely, ECM–receptor interaction pathway and ABC transporters pathway) and six (included Focal adhesion pathway, Phosphatidylinositol signaling system pathway, ECM–receptor interaction pathway, Pentose phosphate pathway, Vascular smooth muscle contraction pathway, and Regulation of actin cytoskeleton pathway) metabolic pathways, respectively (corrected *p*-value < 0.05).

**Figure 7:**
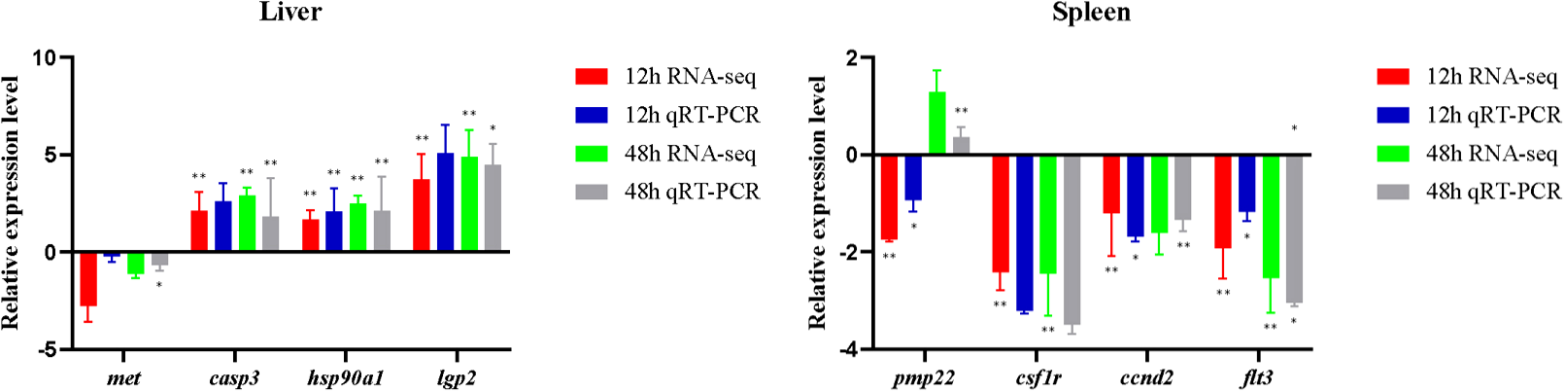
Expression levels of immune-related genes obtained based on RNA-seq and qRT-PCR. Asterisks indicate statistical significance (*p < 0.05) or extremely statistical significance (**p < 0.01).

The expression trend of eight genes was concordant based on the qRT-PCR data and the RNA-seq data, which meant that the sequencing results were reliable. The different genes showed different expression trends after the poly (I:C) challenge (**Figure 7**). Detailed *casp3*, *hsp90a1*, and *lgp2* in the liver were upregulated at 12 h and 48 h after poly (I:C) stimulation, while *met* was downregulated. In the spleen, *csf1r*, *ccnd2*, and *flt3* were downregulated at 12 h and 48 h after poly (I:C) stimulation. Meanwhile, *pmp22* in the spleen was downregulated at 12 h after poly (I:C) stimulation, but upregulated at 48 h after poly (I:C) stimulation.

## 4 Discussion

Disease has been proven to seriously restrict the healthy and sustainable development of aquaculture (33). At present, the use of various chemicals and antibiotics is the essential preventive measure in aquaculture (22, 34, 35). It is worth noting that long-term use of drug in aquaculture may lead to a series of disasters, such as decreased fish immunity, increased pathogen resistance, and drug residues. Immune adjuvants can significantly improve the antigen-specific immune response and vaccine efficacy of fish in a short period of time, so they are gradually applied in the aquaculture (12, 36). For *L. lota* aquaculture, understanding the transcriptional regulatory mechanisms of *L. lota* to immune adjuvants will help to predict the response ability of *L. lota* to viral diseases, and further provide a theoretical basis for exploring disease prevention and control strategies in the *L. lota* aquaculture processes.

### 4.1 High RNA-seq reads accuracy

The present clean RNA-seq reads covers more than 90% of the *L. lota* genome, and the percentage of clean RNA-seq reads with multiple locations on the genome were less than 10%. This means that our sequenced reads were uncontaminated. Meanwhile, a large proportion of reads could be compared to the exon region and a small proportion to the intergenic region, which provided evidence for the integrity of the *L. lota genome* annotation information. Residues from pre-mRNA and intron retention events during AS processes resulted in a small number of reads being compared to the intron region. Additionally, many protein-coding genes (20,646/21,664; 95.30%; 29) can be functionally annotated in at least one protein database. In conclusion, our sequencing reads can provide a basic resource for studying the regulatory mechanisms of *L. lota* to poly (I:C).

### 4.2 Genetic regulatory changes in liver and spleen of *L. lota* exposed to poly (I:C)

#### 4.2.1 DEGs

The expression changes of functional genes may contribute to the efficient response of *L. lota* to poly (I:C) challenge. We found that the expression levels of many functional genes in liver and spleen of *L. lota* were changed in response to poly (I:C) stimulation, which also provided evidence for the immune-inducing properties of poly (I:C) (22, 26). Meanwhile, we also found that the number of DEGs and upregulated genes increased with the duration (12 h to 48 h) of poly (I:C) stimulation in both liver and spleen. This may mean that *L. lota* need to activate more efficient gene expression patterns in response to longer periods of poly (I:C) stimulation. The clustering results show similarity in functional gene expression levels, but did not represent exact phylogenetic relationships of functional genes. However, the clustering results may indirectly confirm that the DEGs we acquired are critical functional genes that respond to poly (I:C) stimulation. It is understandable that functionally similar genes derived from the same tissue show similar expression levels in response to similar stimulation, but the expression levels of the same functional genes in response to the same stimulus will show tissue specificity, which may depend on the stimulus reception time and response ability of tissue. In the present study, eight randomly selected immune-related DEGs (namely, *casp3*, *hsp90a1*, *lgp2*, *met*, *csf1r*, *ccnd2*, *flt3*, and *pmp22*) were further verified by qRT-PCR. Upregulated *casp3* in *L. lota* liver exposed for 12 h and 48 h can cleave and inactivate a variety of important proteins in the cytoplasm, resulting in an imbalance of cellular homeostasis and ultimately leading to apoptosis of damaged liver cells, which contributes to the maintenance of metabolic capacity, structural integrity, and function of the liver stimulated by poly (I:C) (37). The heat shock proteins (HSPs) encoded by the *HSP90a1* is the core component of HSP90-SGT1-RAR1, which has been proven to be an essential signaling component of immune response (38). Accumulating evidence suggests that *lgp2* is a member of the pathogen recognition receptor family and plays a key role in recognizing the innate immune response induced by viral infection (39). Therefore, we boldly speculated that the upregulation of *HSP90a1* and *lgp2* ensures enhanced liver response to poly (I:C) to ensure a rapid response of liver to the invading virus. Additionally, we also found that the expression of *met* in the liver was downregulated, and *met* has been confirmed to be involved in hepatocyte growth factor receptor, which may imply that poly (I:C) stimulation may lead to liver injury in *L. lota* (40). The inhibition of *met* is essential to avoid excessive cell proliferation and maintain normal biological processes in the liver (41). Three functional genes (namely, *csf1r*, *ccnd2*, and *flt3*) in the spleen were downregulated at 12 h and 48 h after poly (I:C) stimulation. *csf1r* may contribute to the inhibition of macrophages, which has a positive effect on the activation of immune responses in damaged tissues (42). It is not clear why *csf1r* decreases its expression, and this could link inhibition of the macrophage-mediated response to a positive effect on the activation of the immune response in damaged tissues. The *ccnd2* gene can inhibit the growth of DNA-damaged cells by regulating the expression of various transcription factors (43). A previous study has confirmed that loss- of-function mutations in the *flt3* promote the development of blood cells, which are particularly important for immune responses, through a compensatory increase in ligand levels (44). Additionally, we also found that *pmp22* was downregulated at 12 h after poly (I:C) stimulation, but upregulated at 48 h after poly (I:C) stimulation. It is worth noting that *pmp22* is a negative regulator that can precisely regulate the cell cycle, and high expression of *pmp22* can often arrest cells in the G_0_/G_1_ phase (45). In conclusion, the expression levels of some functional genes in liver and spleen of *L. lota* were changed, thus guaranteeing the normal metabolism, tissue structure, and life state of poly (I:C)-stimulated *L. lota*. We do not deny that there are more critical functional genes that have not attracted our attention or carried out qRT-PCR detection, which is also needed for future research.

#### 4.2.2 Physiological regulation mechanisms

The physiological regulation mechanisms of poly (I:C)-stimulated *L. lota* showed tissue and time specificity (46). The DEGs of *L. lota* exposed to poly (I:C) stimulation were discovered to have protein-regulated functions. Undoubtedly, *L. lota* stimulated by poly (I:C) is energy-consuming (47); thus, changes in protein regulation may have provided energy for cell survival and other life activities (48). A previous study has also confirmed that fishes have a much higher protein requirement than other vertebrates because they are less adept at using lipid and carbohydrate for energy, which may be more pronounced in diseased conditions (48). We also found that some DEGs were involved in the regulation of threonine, which may contribute to the improvement of amino acid utilization (49) and further improve the energy production of *L. lota* stimulated by poly (I:C). Some DEGs have been hypothesized to be associated with cellular homeostasis and immune cell integrity. This may imply that *L. lota* can reduce the toxic effects of poly (I:C) by maintaining the cytoskeletal integrity of liver and spleen (50). We also found that some DEGs were involved in the ion transport and cell death. In fact, toxic substances may require specific carriers (such as organic anions) to enter cells and cause cell death (51), and thus *L. lota* may inhibit the entry of poly (I:C) into cells by these ion transport-related genes.

The upregulated genes in both the liver and spleen were significantly enriched in three poly (I:C) detection and antiviral innate immunity-related pathways, namely, Herpes simplex infection pathway (52), Proteasome pathway (53), and RIG-I-like receptor signaling pathway (54). Except for three shared pathways, upregulated genes were also significantly enriched in the Apoptosis pathway, Toll-like receptor (TLR) signaling pathway, NOD-like receptor (NLR) signaling pathway, p53 signaling pathway, JAK-STAT signaling pathway, and Drug metabolism - other enzymes pathway. Apoptosis is an autonomous and orderly cell suicide process controlled by genes, which is necessary for the regulation of immune function and maintenance of tissue stability (55, 56). The disorder of apoptosis will lead to a series of pathological changes (55, 56). We hypothesized that the *L. lota* stimulated by poly (I:C) activated the apoptosis pathway, and the damaged cells thus entered apoptosis and were eliminated. Pattern recognition receptor (PRR)-related pathways (including the TLR and NLR signaling pathway) have also been annotated. PRRs are the bridge between innate and acquired immunity, and their activators can be used as immunoadjuvants (57–59). Poly (I:C) has been shown to activate the PRR signaling pathway after binding to PRRs and then exerting innate immunity (60). In fact, the activation of the PRR signaling pathways may also contribute to apoptosis of *L. lota*-damaged liver or spleen cells (61). The significant enrichment of upregulated genes in the p53 signaling pathway may also be applied to enable poly (I:C)-damaged cells to perform DNA repair or accelerate apoptosis (62). It is well established that organisms invaded by viruses can regulate cell cycle arrest, DNA repair, and apoptosis by regulating *p53* gene and p53 protein (63, 64). Additionally, the JAK-STAT signaling pathway as an important cytokine-stimulated signal transduction pathway was also activated by poly (I:C) stimulation (65). The JAK-STAT signaling pathway has been confirmed to be involved in the immune regulation, cell proliferation, differentiation, and apoptosis of other teleost fishes [i.e., *Carassius auratus gibelio* (66), *Tachysurus fulvidraco* (67), and *Sebastiscus marmoratus* (68)] stimulated by poly (I:C). Considering that the virus mainly hides in cells and replicates and spreads through cellular metabolism (67), we hypothesized that *L. lota* may initiate the cellular apoptotic process to limit the contagion of poly (I:C) in the liver or spleen (68). Some upregulated genes also significantly enriched the Drug metabolism - other enzymes pathway. It was not difficult to find that poly (I:C) stimulation changed the enzyme catalytic capacity of liver or spleen in *L. lota*. Enzymes have been confirmed to be involved in a variety of biological processes in diseased fish, including cell proliferation and death, cell migration, cytoskeleton dynamics, cell cycle regulation, and regulation of numerous signaling pathways (69, 70). Meanwhile, some enzymes, such as proteasome and lysozyme, are immune molecules secreted by immune cells, which can improve and enhance macrophage phagocytosis ability and thus play a critical role in the defense against pathogens in fishes (53, 71–73).

The downregulated genes in both liver and spleen were significantly enriched in the Focal adhesion pathway, ECM–receptor interaction pathway, Vascular smooth muscle contraction pathway, and Regulation of actin cytoskeleton pathway. Focal adhesion kinase (FAK) is a critical enzyme in the Focal adhesion pathway, which can integrate viral signals from outside the cell and regulate the activity of downstream molecules, thereby controlling cell metabolism, proliferation, and even cell fate (74). FAK has also been shown to degrade p53 protein through the ubiquitination pathway and prevent cellular apoptosis (74). Therefore, there is reason to believe that activation of the Focal adhesion pathway may hinder the clearance of *L. lota*-damaged cells. The ECM–receptor interaction pathway, Vascular smooth muscle contraction pathway, and Regulation of actin cytoskeleton pathway were confirmed to be involved in cell migration, which is required for immune surveillance, and tissue repair and regeneration (75, 76). The virus-invaded cell migration drives progression of fish diseases, and thus, we hypothesized that the downregulation of genes related to the three pathways mentioned above may contribute to the reduction of the poly (I:C) invasion of *L. lota* liver or spleen cells. Additionally, the downregulated genes in the liver were also significantly enriched in some specific pathways, including the Phosphatidylinositol (PI) signaling system pathway, Inositol phosphate (IP) metabolism pathway, Notch signaling pathway, Lysine degradation pathway, Cytokine–cytokine receptor interaction pathway, ABC transporters pathway, Glycerolipid metabolism pathway, and Insulin signaling pathway. Previous studies have demonstrated that the PI signaling system pathway and IP metabolism pathway can reduce the proliferation ability and induce apoptosis of DNA-damaged cells by reducing the concentration and activity of IP of PI kinases (77). This is mainly because PI and IP kinases can cause the release of intracellular calcium, which is the regulator of intracellular messenger and cellular activity and is critical for cell survival (78, 79). The Notch signaling pathway can inhibit the inflammatory response of macrophages induced by TLRs (80, 81), and lead to the impairment of M1 type activation of macrophages (82). Therefore, inhibition of the Notch signaling pathway is valuable for the activation of macrophages in *L. lota* liver. Lysine is a non-specific bridging molecule that connects antigens to T cells (83). The lack of lysine reduces cytokine synthesis, thereby inhibiting lymphocyte proliferation and reducing cell-mediated immune response (84). In this study, the downregulation of the Lysine degradation pathway and Cytokine–cytokine receptor interaction pathway may safeguard lysine and cytokine content and ultimately enhance the liver cell-mediated immune response of *L. lota*. ABC transporters perform various physiological functions such as resistance to foreign invasion, antigen presentation, and lipid transport (85). We also found that the downregulated genes in the liver were significantly enriched in the ABC transporter pathway, but the immune response of this pathway is still worth further investigation in future studies. Additionally, the downregulated genes in poly (I:C)-stimulated *L. lota* liver were also significantly enriched in the lipid and glucose metabolism-related pathways (Glycerolipid metabolism pathway and Insulin signaling pathway). Liver is not only an important immune organ, but also a very important metabolic organ, which is very important for the balance of glucose and lipid metabolism and the maintenance of energy homeostasis (86). Meanwhile, it should be noted that the inhibited Insulin signaling pathway can stimulate gluconeogenesis and fatty acid oxidation, which are critical for the release of large amounts of energy, and help to fuel the immune response of *L. lota* liver cells.

In conclusion, functional genes and their products of the liver and spleen exerted their effects to regulate the physiological functions of *L. lota* stimulated by poly (I:C), and finally ensured their survival. According to the inferred potential function of poly (I:C)-stimulated DEGs, we have proposed management measures for *L. lota* attacked by viruses, as follows: the regulation of the breeding environment is the first development, which not only is conducive to eliminating or alleviating the stress response of virus-infected *L. lota*, but also can increase the efficacy of drugs; protein (especially lysine) supplementation in the diet is also necessary because *L. lota* consume energy to deal with the virus; and lysine supplementation will aid in cytokine synthesis and proliferation of lymphocytes required for the immune response.

## 5 Conclusion

Poly (I:C) as an immune adjuvant can be used to explore the viral immune responses of *L. lota*. In this study, RNA-seq was used for the first time to determine the transcriptome of liver and spleen of *L. lota* stimulated by poly (I:C) to explore the genetic regulatory mechanisms of *L. lota* to immune adjuvant. We found that the liver and spleen of *L. lota* showed different responses to poly (I:C) stimulation, which was manifested at the levels of DEGs and related biological functions. Meanwhile, we suspected that the stress of liver and spleen of *L. lota* is serious with the extension of poly (I:C) stimulation time (12 h to 48 h). Based on the annotation information of DEGs, we hypothesized that *L. lota* could initiate apoptosis of DNA-damaged cells to inhibit poly (I:C) propagation in the liver and spleen. Meanwhile, some enzymes that enhance immune effects and metabolic mechanisms that provide energy have also been found to be necessary for *L. lota* to cope with the poly (I:C) challenge. This study provides basic resources for exploring the regulatory mechanisms of *L. lota* to poly (I:C) and other immune adjuvants. Furthermore, we expect that the present study can provide valuable information for future disease prevention in *L. lota* artificial culture.

## Data availability statement

The datasets presented in this study can be found in online repositories. The names of the repository/repositories and accession number(s) can be found in the article/**Supplementary Material**.

## Ethics statement

The animal studies were approved by the Institutional Animal Care and Use Committee of Yantai University. The studies were conducted in accordance with the local legislation and institutional requirements. Written informed consent was obtained from the owners for the participation of their animals in this study.

## Author contributions

FL: Data curation, Formal Analysis, Methodology, Software, Writing – original draft. YZ: Investigation, Methodology, Writing – original draft. AX: Data curation, Investigation, Writing – original draft. TG: Conceptualization, Funding acquisition, Resources, Supervision, Visualization, Writing – review & editing.

## Supplementary material

Supplementary file 3 Distribution of RNA-seq reads of each sample in different regions of the reference genome.

Supplementary file 4 Density distribution of RNA-seq reads of each sample on chromosome.

Supplementary file 5 The DEGs among four experimental pairs (L_12po_-vs-L_12PBS_, L_48po_-vs-L_48PBS_, S_12po_-vs-S_12PBS_, and S_48po_-vs-S_48PBS_).

Supplementary file 6 The common DEGs among four experimental pairs.

Supplementary file 7 The significantly enriched GO terms of the up- and downregulated genes in the liver and spleen.

Supplementary file 8 The significantly enriched KEGG pathways of the up- and downregulated genes in the liver and spleen.
