# Supplementary figures and images for: Transcriptional responses of liver and spleen in Lota lota to polyriboinosinic polyribocytidylic acid
Source: Front Immunol. 2023 Oct 13;14:1272393. doi: 10.3389/fimmu.2023.1272393 (PMC10611466; doi:10.3389/fimmu.2023.1272393)

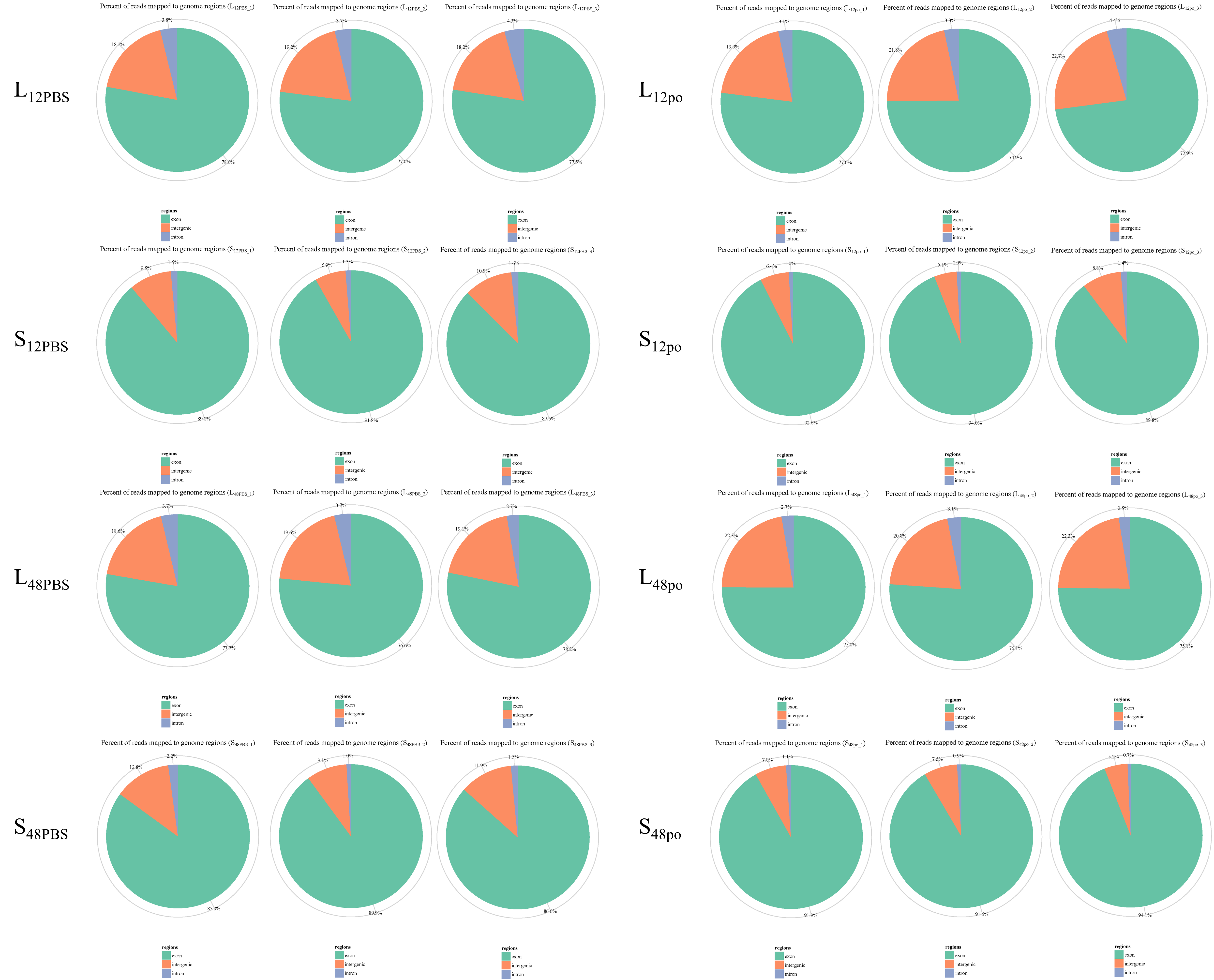

Supplement: Supplementary file 1 [file Image_1.png]

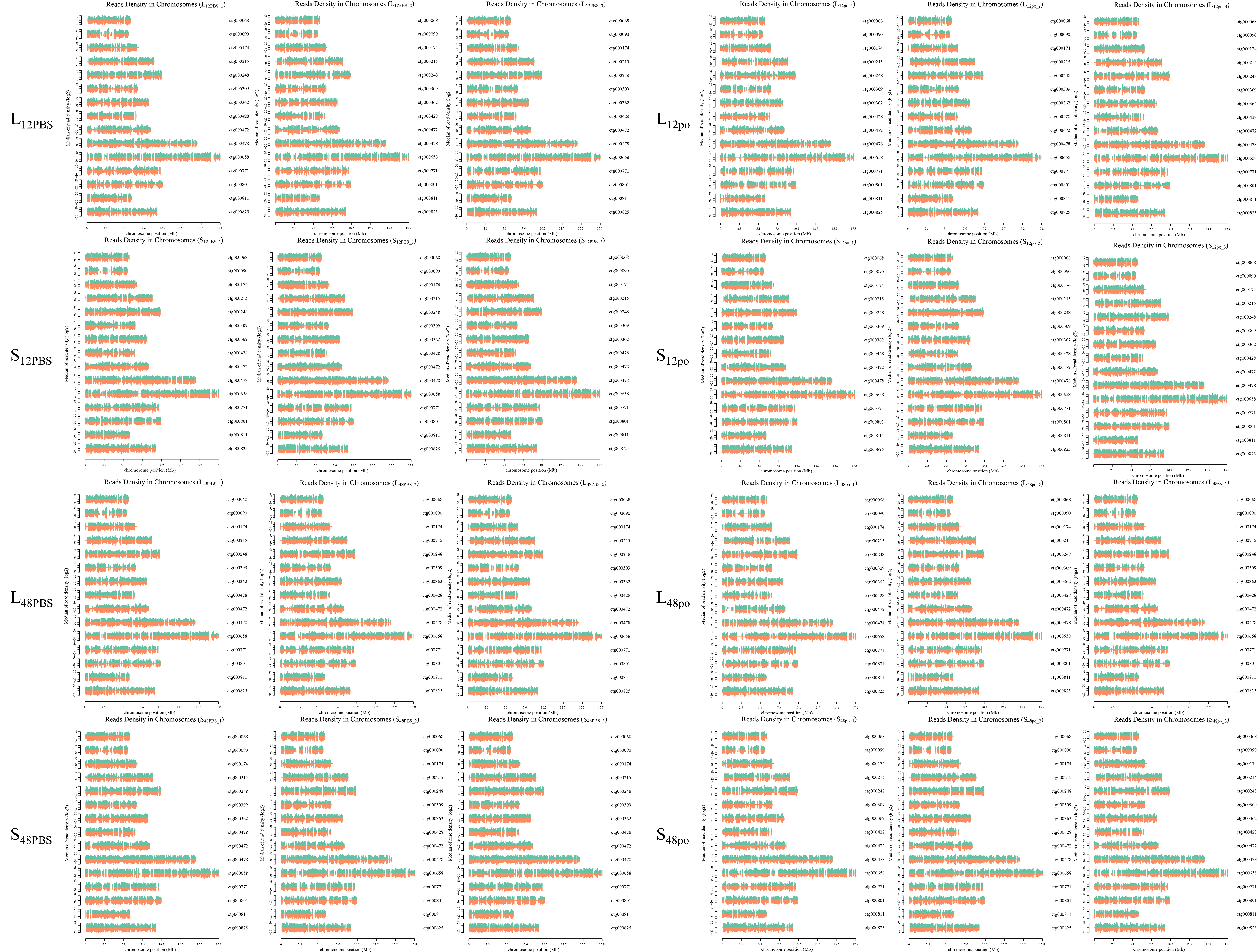

Supplement: Supplementary file 2 [file Image_2.png]
