# Supplementary material for: Transcriptional responses of liver and spleen in Lota lota to polyriboinosinic polyribocytidylic acid
Source: Front Immunol. 2023 Oct 13;14:1272393. doi: 10.3389/fimmu.2023.1272393 (PMC10611466; doi:10.3389/fimmu.2023.1272393)
Supplement: Supplementary file 3 — Distribution of RNA-seq reads of each sample in different regions of the reference genome. [file Table_1.docx]

Supplementary file 1. Summary of RNA-seq reads output quality.

| **Group** | **Sample name** | **Raw reads** | **Clean reads** | **Clean bases** | **Error rate(%)** | **Q20(%)** | **Q30(%)** | **GC content(%)** |
| --- | --- | --- | --- | --- | --- | --- | --- | --- |
| L_12PBS_ | L_12PBS_1_ | 67777774 | 65794196 | 9.87GB | 0.03 | 96.33 | 90.86 | 51.74 |
|  | L_12PBS_2_ | 70903062 | 68896004 | 10.33GB | 0.03 | 96.47 | 91.12 | 51.53 |
|  | L_12PBS_3_ | 73060998 | 70398272 | 10.56GB | 0.03 | 96.36 | 90.92 | 51.95 |
| S_12PBS_ | S_12PBS_1_ | 81286996 | 79275204 | 11.89GB | 0.03 | 96.72 | 91.63 | 53.42 |
|  | S_12PBS_2_ | 74405158 | 71612526 | 10.74GB | 0.03 | 96.46 | 91.11 | 53.62 |
|  | S_12PBS_3_ | 68167418 | 65747202 | 9.86GB | 0.03 | 96.66 | 91.54 | 52.13 |
| L_48PBS_ | L_48PBS_1_ | 65911510 | 63479602 | 9.52GB | 0.03 | 96.50 | 91.17 | 51.21 |
|  | L_48PBS_2_ | 61780796 | 59264970 | 8.89GB | 0.03 | 96.39 | 90.98 | 51.07 |
|  | L_48PBS_3_ | 67139506 | 64760884 | 9.71GB | 0.03 | 96.55 | 91.25 | 51.32 |
| S_48PBS_ | S_48PBS_1_ | 68231476 | 65863240 | 9.88GB | 0.03 | 96.61 | 91.40 | 51.92 |
|  | S_48PBS_2_ | 63399412 | 60676992 | 9.10GB | 0.03 | 96.57 | 91.33 | 52.23 |
|  | S_48PBS_3_ | 67967858 | 65281574 | 9.79GB | 0.03 | 96.41 | 91.05 | 51.79 |
| L_12po_ | L_12po_1_ | 61582668 | 59211538 | 8.88GB | 0.03 | 96.67 | 91.48 | 51.28 |
|  | L_12po_2_ | 65123058 | 62747606 | 9.41GB | 0.03 | 96.58 | 91.31 | 50.84 |
|  | L_12po_3_ | 63393330 | 61034242 | 9.16GB | 0.03 | 96.33 | 90.82 | 50.49 |
| S_12po_ | S_12po_1_ | 53139112 | 50944696 | 7.64GB | 0.03 | 96.76 | 91.70 | 52.50 |
|  | S_12po_2_ | 74255656 | 70986766 | 10.65GB | 0.03 | 96.79 | 91.76 | 54.41 |
|  | S_12po_3_ | 70533518 | 66679822 | 10.00GB | 0.03 | 96.73 | 91.66 | 51.97 |
| L_48po_ | L_48po_1_ | 53815122 | 52360314 | 7.85GB | 0.03 | 96.68 | 91.45 | 50.79 |
|  | L_48po_2_ | 62312060 | 60616520 | 9.09GB | 0.03 | 96.51 | 91.17 | 50.96 |
|  | L_48po_3_ | 58721218 | 56524896 | 8.48GB | 0.03 | 96.59 | 91.27 | 51.06 |
| S_48po_ | S_48po_1_ | 64655370 | 62352080 | 9.35GB | 0.03 | 96.71 | 91.59 | 51.97 |
|  | S_48po_2_ | 63166178 | 61232940 | 9.18GB | 0.03 | 96.60 | 91.38 | 52.80 |
|  | S_48po_3_ | 56841412 | 55030122 | 8.25GB | 0.03 | 96.62 | 91.41 | 52.78 |
