# Supplementary material for: Transcriptional responses of liver and spleen in Lota lota to polyriboinosinic polyribocytidylic acid
Source: Front Immunol. 2023 Oct 13;14:1272393. doi: 10.3389/fimmu.2023.1272393 (PMC10611466; doi:10.3389/fimmu.2023.1272393)
Supplement: Supplementary file 4 — Density distribution of RNA-seq reads of each sample on chromosome. [file Table_2.docx]

Supplementary file 2. Comparison results of clean RNA-seq reads with reference genome.

| **Group** | **Sample name** | **Total reads** | **Total mapped** | **Multiple mapped** | **Uniquely mapped** | **Reads map to '+'** | **Reads map to '-'** | **Non-splice reads** | **Splice reads** |
| --- | --- | --- | --- | --- | --- | --- | --- | --- | --- |
| L_12PBS_ | L_12PBS_1_ | 65794196 | 60013591 (91.21%) | 5530098 (8.41%) | 54483493 (82.81%) | 27199847 (41.34%) | 27283646 (41.47%) | 23415556 (35.59%) | 31067937 (47.22%) |
|  | L_12PBS_2_ | 68896004 | 62982290 (91.42%) | 5836005 (8.47%) | 57146285 (82.95%) | 28528766 (41.41%) | 28617519 (41.54%) | 25009392  (36.30%) | 32136893 (46.65%) |
|  | L_12PBS_3_ | 70398272 | 64264173 (91.29%) | 6531558 (9.28%) | 57732615 (82.01%) | 28808383 (40.92%) | 28924232 (41.09%) | 24649292 (35.01%) | 33083323 (46.99%) |
| S_12PBS_ | S_12PBS_1_ | 79275204 | 72484301 (91.43%) | 4947932 (6.24%) | 67536369 (85.19%) | 33574342 (42.35%) | 33962027 (42.84%) | 23296816 (29.39%) | 44239553 (55.81%) |
|  | S_12PBS_2_ | 71612526 | 65495332 (91.46%) | 4161495 (5.81%) | 61333837 (85.65%) | 30458091 (42.53%) | 30875746 (43.12%) | 19720295 (27.54%) | 41613542 (58.11%) |
|  | S_12PBS_3_ | 65747202 | 59723044 (90.84%) | 3167370 (4.82%) | 56555674 (86.02%) | 28126963 (42.78%) | 28428711 (43.24%) | 21885508 (33.29%) | 34670166 (52.73%) |
| L_48PBS_ | L_48PBS_1_ | 63479602 | 57841094 (91.12%) | 5411801 (8.53%) | 52429293 (82.59%) | 26177200 (41.24%) | 26252093 (41.36%) | 22592667 (35.59%) | 29836626 (47.00%) |
|  | L_48PBS_2_ | 59264970 | 53881197 (90.92%) | 4625958 (7.81%) | 49255239 (83.11%) | 24609424 (41.52%) | 24645815 (41.59%) | 21263707 (35.88%) | 27991532 (47.23%) |
|  | L_48PBS_3_ | 64760884 | 59077462 (91.22%) | 5139974 (7.94%) | 53937488 (83.29%) | 26907667 (41.55%) | 27029821 (41.74%) | 22884259 (35.34%) | 31053229 (47.95%) |
| S_48PBS_ | S_48PBS_1_ | 65863240 | 59509491 (90.35%) | 3996583 (6.07%) | 55512908 (84.29%) | 27608932 (41.92%) | 27903976 (42.37%) | 22330093  (33.90%) | 33182815 (50.38%) |
|  | S_48PBS_2_ | 60676992 | 54867432 (90.43%) | 3115490 (5.13%) | 51751942 (85.29%) | 25711060 (42.37%) | 26040882 (42.92%) | 18748543  (30.90%) | 33003399 (54.39%) |
|  | S_48PBS_3_ | 65281574 | 58878903 (90.19%) | 4425389 (6.78%) | 54453514 (83.41%) | 27076082 (41.48%) | 27377432 (41.94%) | 22167591 (33.96%) | 32285923 (49.46%) |
| L_12po_ | L_12po_1_ | 59211538 | 54044475 (91.27%) | 5513579 (9.31%) | 48530896 (81.96%) | 24205970 (40.88%) | 24324926 (41.08%) | 20908027 (35.31%) | 27622869 (46.65%) |
|  | L_12po_2_ | 62747606 | 57066711 (90.95%) | 5896300  (9.40%) | 51170411 (81.55%) | 25540566 (40.70%) | 25629845 (40.85%) | 23133505 (36.87%) | 28036906 (44.68%) |
|  | L_12po_3_ | 61034242 | 55295098 (90.60%) | 5362608 (8.79%) | 49932490 (81.81%) | 24931695 (40.85%) | 25000795 (40.96%) | 22794212 (37.35%) | 27138278 (44.46%) |
| S_12po_ | S_12po_1_ | 50944696 | 46147013 (90.58%) | 2751792  (5.40%) | 43395221 (85.18%) | 21571421 (42.34%) | 21823800 (42.84%) | 15526617 (30.48%) | 27868604 (54.70%) |
|  | S_12po_2_ | 70986766 | 65776480 (92.66%) | 4830954 (6.81%) | 60945526 (85.85%) | 30317725 (42.71%) | 30627801 (43.15%) | 17520274 (24.68%) | 43425252 (61.17%) |
|  | S_12po_3_ | 66679822 | 60122359 (90.17%) | 3452374 (5.18%) | 56669985 (84.99%) | 28156251 (42.23%) | 28513734 (42.76%) | 20975499 (31.46%) | 35694486 (53.53%) |
| L_48po_ | L_48po_1_ | 52360314 | 47749850 (91.19%) | 4212607 (8.05%) | 43537243 (83.15%) | 21717756 (41.48%) | 21819487 (41.67%) | 18044614 (34.46%) | 25492629 (48.69%) |
|  | L_48po_2_ | 60616520 | 54930533 (90.62%) | 4868067 (8.03%) | 50062466 (82.59%) | 25040315 (41.31%) | 25022151 (41.28%) | 20083269 (33.13%) | 29979197 (49.46%) |
|  | L_48po_3_ | 56524896 | 51435830 (91.00%) | 4784853 (8.47%) | 46650977 (82.53%) | 23302057 (41.22%) | 23348920 (41.31%) | 19392049 (34.31%) | 27258928 (48.22%) |
| S_48po_ | S_48po_1_ | 62352080 | 56006890 (89.82%) | 3268219 (5.24%) | 52738671 (84.58%) | 26166963 (41.97%) | 26571708 (42.62%) | 19043719 (30.54%) | 33694952 (54.04%) |
|  | S_48po_2_ | 61232940 | 55840731 (91.19%) | 3500652 (5.72%) | 52340079 (85.48%) | 26068415 (42.57%) | 26271664 (42.90%) | 18223255 (29.76%) | 34116824 (55.72%) |
|  | S_48po_3_ | 55030122 | 49997969 (90.86%) | 3231246 (5.87%) | 46766723 (84.98%) | 23239181 (42.23%) | 23527542 (42.75%) | 15951798 (28.99%) | 30814925 (56.00%) |
|  | Mean | 63365509 | 57643010  (90.97%) | 4523454  (7.14%) | 53119556  (83.83%) | 26460211  (41.76%) | 26659345  (42.07%) | 20815023  (32.85%) | 32304533  (50.99%) |
